# Broadband stimulated Raman scattering microscopy with wavelength‐scanning detection

**DOI:** 10.1002/jrs.5816

**Published:** 2020-01-03

**Authors:** Alejandro De la Cadena, Carlo M. Valensise, Marco Marangoni, Giulio Cerullo, Dario Polli

**Affiliations:** ^1^ IFN‐CNR, Dipartimento di Fisica Politecnico di Milano Milano Italy

**Keywords:** coherent Raman spectroscopy, hyperspectral microscopy, nonlinear optical microscopy, optical parametric oscillators, stimulated Raman scattering

## Abstract

We introduce a high‐sensitivity broadband stimulated Raman scattering (SRS) setup featuring wide spectral coverage (up to 500 cm^−1^) and high‐frequency resolution (≈20 cm^−1^). The system combines a narrowband Stokes pulse, obtained by spectral filtering an Yb laser, with a broadband pump pulse generated by a home‐built optical parametric oscillator. A single‐channel lock‐in amplifier connected to a single‐pixel photodiode measures the stimulated Raman loss signal, whose spectrum is scanned rapidly using a galvanometric mirror after the sample. We use the in‐line balanced detection approach to suppress laser fluctuations and achieve close to shot‐noise‐limited sensitivity. The setup is capable of measuring accurately the SRS spectra of several solvents and of obtaining hyperspectral data cubes consisting in the broadband SRS microscopy images of polymer beads test samples as well as of the distribution of different biological substances within plant cell walls.

## Graphical abstract

We introduce a high‐sensitivity broadband stimulated Raman scattering (SRS) setup featuring wide spectral coverage (up to 500 cm^−1^) and high‐frequency resolution (≈20 cm^−1^). A single‐channel lock‐in amplifier connected to a single‐pixel photodiode measures the Stimulated Raman loss signal, whose spectrum is scanned rapidly using a galvanometric mirror after the sample. We use the in‐line balanced detection approach to suppress laser fluctuations. We present the SRS spectra of solvents as well as microspectroscopy data of reference microstructures and plant samples.

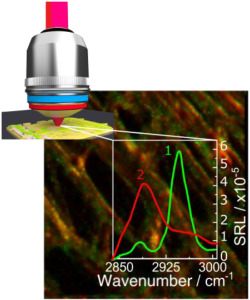

## 1 INTRODUCTION

Coherent Raman scattering (CRS) microscopy^1^, ^2^, ^3^ allows label‐free identification of molecules based on their intrinsic vibrational response, which provides a fingerprint of their chemical structure. CRS exploits the resonant third‐order nonlinear optical signal generated in a sample by interaction with two light pulses, the pump (at frequency ω_p_) and the Stokes (at frequency ω_S_), when their frequency detuning ω_p_–ω_S_ matches a vibrational frequency Ω of the molecule. In coherent anti‐Stokes Raman scattering,^4^, ^5^, ^6^, ^7^ the response is read at the anti‐Stokes frequency ω_aS_ = ω_p_ + Ω, whereas in stimulated Raman scattering (SRS),^8^, ^9^, ^10^ the nonlinear signal is imprinted on the pump/Stokes pulses themselves, in the form of Stokes pulse amplification (stimulated Raman gain, SRG) or pump pulse attenuation (stimulated Raman loss, SRL). Coherent anti‐Stokes Raman scattering suffers from the superposition with a frequency‐independent nonresonant background, which often distorts and masks the resonant signal of interest^11^, ^12^; SRS, on the other hand, is almost free from nonlinear background and directly measures the resonant signal, making it the CRS microscopy technique of choice.^13^, ^14^, ^15^, ^16^, ^17^

The main technical challenge of SRS lies in the requirement to measure the very weak SRG/SRL signal (of the order of 10^−5^ ÷ 10^‐4^) sitting on top of the fluctuating Stokes/pump background, which calls for the development of a high‐sensitivity detection chain. In single‐frequency SRS, which uses narrowband (≈10–20 cm^−1^) pump and Stokes pulses and detects the signal for a specific detuning Ω, high sensitivity is typically achieved by combining high‐frequency (up to tens of MHz) modulation of the pump/Stokes beam and synchronous detection of the SRG/SRL signal with a lock‐in amplifier, leading to acquisition speeds up to the video rate.^18^ However, its information content is limited and in many cases not sufficient to distinguish the different components within complex heterogeneous systems containing several spectrally overlapped chemical species. This has motivated intense research in the development of broadband SRS techniques, which have the goal of recording, for each pixel of the image, a complete SRS spectrum.^19^ They can be classified into two categories^19^: hyperspectral SRS, in which the spectrum is acquired sequentially by rapidly scanning the pump–Stokes frequency detuning; multiplex SRS, in which the SRS spectrum is acquired in parallel by an array of photodetectors.

The simplest approach to hyperspectral SRS relies on the use of two narrowband lasers, whose frequency detuning is rapidly scanned. Ozeki et al.^20^, ^21^ coupled a picosecond Ti:sapphire laser to an electronically synchronized Yb:fiber laser incorporating a tunable band pass filter, whereas Kong et al.^22^ inserted an electro‐optic Lyot filter in the cavity of an optical parametric oscillator (OPO) to rapidly tune its output. Alternatively, rapid frequency tuning in hyperspectral SRS can be achieved using broadband pump and Stokes pulses and applying the spectral focusing (SF)^23^, ^24^, ^25^ technique. In SF, a large frequency chirp is imparted to both the pump and the Stokes pulses so that their instantaneous frequency difference remains constant and matches a specific vibrational frequency. The instantaneous frequency difference can then be swiftly tuned by changing the pump–Stokes delay using a rapid‐scanning optical delay line. Liao et al.^26^ inserted a 12‐kHz resonant galvanometric mirror in the Fourier plane of a 4f pulse shaper to measure spectra with pixel dwell times of 83 μs, whereas Alshaykh et al.^27^ used an acousto‐optically programmable dispersive filter to achieve an even higher pixel scan rate of 30 kHz. Laptenok et al.^17^ employed a dual approach: A narrowband (8 cm^−1^) acousto‐optical tunable filter scanned the laser pulse spectral bandwidth (about 150 cm^−1^) for every selected laser central wavelength; subsequently, the laser central wavelength was changed and the scan repeated. In this way, they could cover the entire Raman vibrational region from the fingerprint to the C─H stretch without the need to realign the beam path or to change the optical setup.

Multiplex SRS combines a narrowband pump (Stokes) with a broadband Stokes (pump) pulse and measured the SRG (SRL) simultaneously at all Stokes (pump) wavelengths. Its technical challenge resides in the development of a multichannel detection chain with a sensitivity comparable with that achieved in single‐frequency SRS. Optical multichannel analyzers, which consist of a dispersive spectrometer coupled to a linear detector array, can only work at repetition rates up to 20 kHz,^28^ and their sensitivity is limited by shot noise, which in turn is dictated by the saturation charge of the detectors. Multichannel lock‐in amplifiers were first introduced by Kobayashi and coworkers^29^, ^30^ but only at low modulation frequencies, which severely limited their sensitivity. The best results were obtained by Liao et al.,^31^ who used a lock‐in‐free detector based on an array of 16 tuned amplifiers, consisting of active LC bandpass filters tuned to the modulation frequency, to record an SRS spectrum in a time as short as 32 μs. Alternative approaches to multiplex SRS, such as Fourier‐transform SRS^32^ and photonic time stretch SRS,^33^ are promising but require complex experimental setups.

In this paper, we present a high‐sensitivity broadband SRS setup combining wide spectral coverage (up to 500 cm^‐1^), high‐frequency resolution (≈20 cm^−1^). The system is a hybrid between multiplex and hyperspectral SRS, as it uses a narrowband Stokes pulse synchronized with a broadband pump pulse and employs a galvanometric mirror after the sample to scan rapidly the SRL detection frequency in a high‐sensitivity single‐channel lock‐in detection chain. We use the in‐line balanced detection (IBD)^34^ approach, previously introduced by our group, to suppress the laser fluctuations and achieve a nearly shot‐noise‐limited sensitivity. Our configuration is remarkably simple to be implemented, does not suffer from bandwidth limitations, and can be straightforwardly up‐scaled in acquisition speed by optimizing the galvanometric scanner. As a first step in the characterization and validation of our system, we measured SRS spectra of reference samples whose vibrational response has been systematically investigated with spontaneous Raman and reported elsewhere. The results obtained with our SRS system were cross‐validated with such literature reports. We observed that the widths, Raman bands, and ratios of the spectra obtained with the SRS setup described in the present contribution match very well those from literature.

## 2 EXPERIMENTAL SETUP AND RESULTS

**Figure 1:**
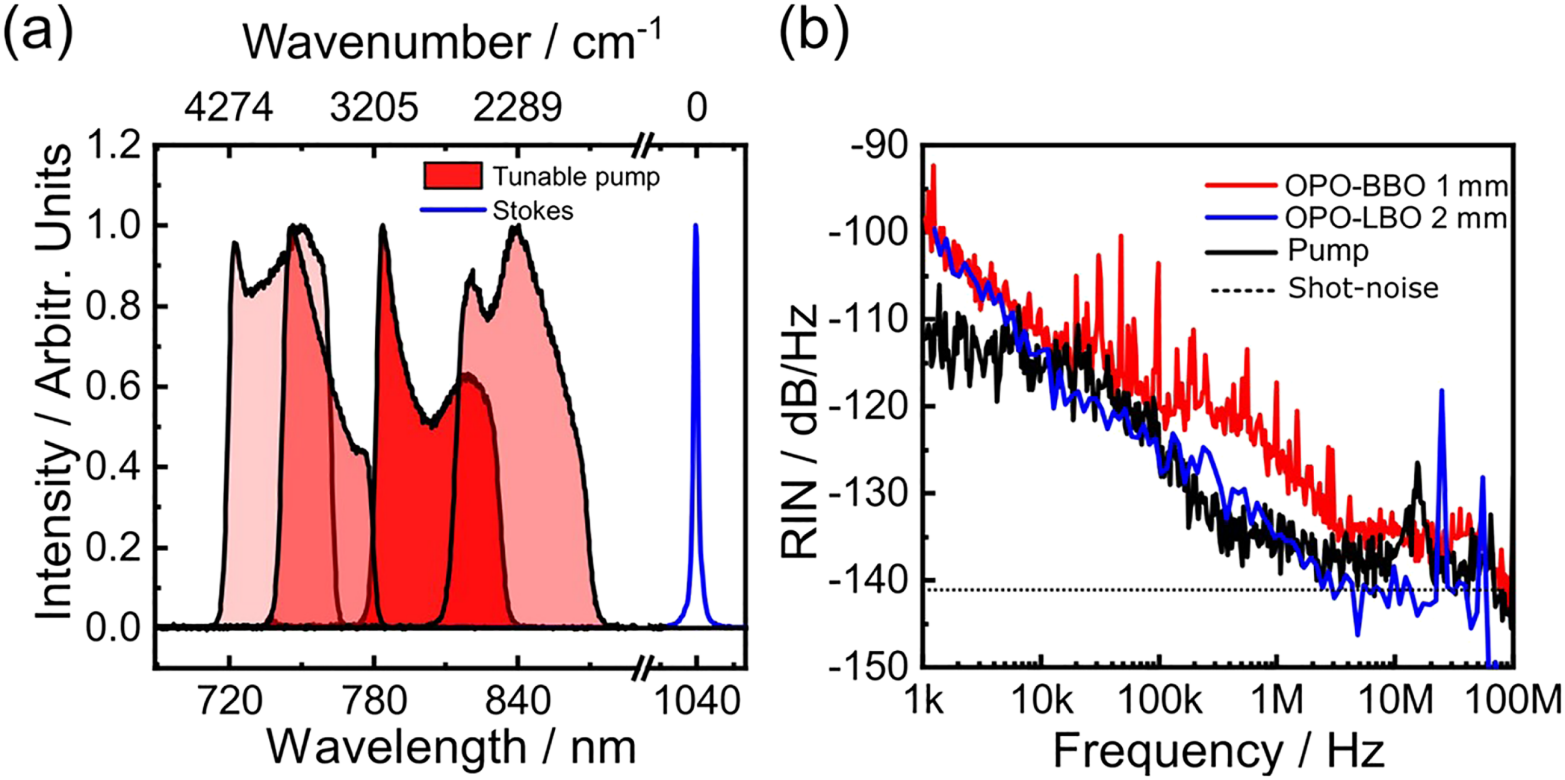
(a) Spectra of the OPO at different cavity lengths, covering the 715–870‐nm wavelength range, and of the Yb:fiber laser frequency filtered by an etalon. (b) Relative intensity noise of the Yb:fiber laser and the OPO with BBO and with LBO crystal. The dashed line indicates the shot‐noise limit of the OPO based on the LBO crystal. BBO, β‐barium‐borate; LBO, lithium triborate; OPO, optical parametric oscillator

Our broadband SRS system starts with a commercial mode‐locked Yb:fiber laser (Coherent Fidelity) producing 140‐fs pulses at 1,040 nm with 10 W average power and 80‐MHz repetition rate. A 4‐W fraction of the power is spectrally filtered by an etalon (SLS optics) to generate narrowband (≈20‐cm^−1^ bandwidth, 1‐ps full width at half maximum duration) Stokes pulses with 1.1 W average power; the remaining 6 W are frequency doubled and used to pump an OPO, which generates the broadband pump pulses. The OPO is built according to the design reported in Coluccelli et al.,^35^ but modified by replacing the 1‐mm‐thick β‐barium‐borate (BBO) nonlinear crystal with a 2‐mm‐thick lithium triborate (LBO) cut for Type‐I phase matching (*θ* = 90°, *φ* = 9.8°). With respect to BBO, the LBO crystal offers a higher acceptance angle and negligible walk‐off,^36^ providing higher effective interaction length and therefore higher parametric gain.^37^ In this way, the OPO cavity requires less pump power, reducing the thermal load on the nonlinear crystal and resulting in an increased stability of the OPO output. The OPO generates pulses with up to 100‐mW average power and spectrum tunable across the 715–870 nm wavelength range by controlling the cavity length with an amplified piezoelectric actuator (APF710, Thorlabs). Figure 1a shows a sequence of OPO spectra, with in excess of 500 cm^−1^ bandwidth, which span the 1,813–4,370 cm^−1^ frequency detuning with respect to the 1,040‐nm Stokes pulse thus covering the whole high‐frequency C─H stretching Raman band, which provides, in many cases, sufficient information for distinguishing different components of cells and tissues, such as DNA, lipids, and proteins, making our broadband SRS system valuable for many biological applications.^38^ The relative intensity noise (RIN), which is a critical parameter for the SRS application, was measured with a high‐frequency digital lock‐in amplifier (LIA, model HF2LI, Zurich Instruments). Figure 1(b) compares RIN spectra of the commercial Yb:fiber pump laser (black curve) with those of the home‐made OPO equipped with the BBO (red curve) and the LBO (blue curve) crystals. The figure demonstrates the quieter performance of the LBO‐based OPO, which, at frequencies higher than 10 kHz, has a RIN comparable with that of the pump laser.

**Figure 2:**
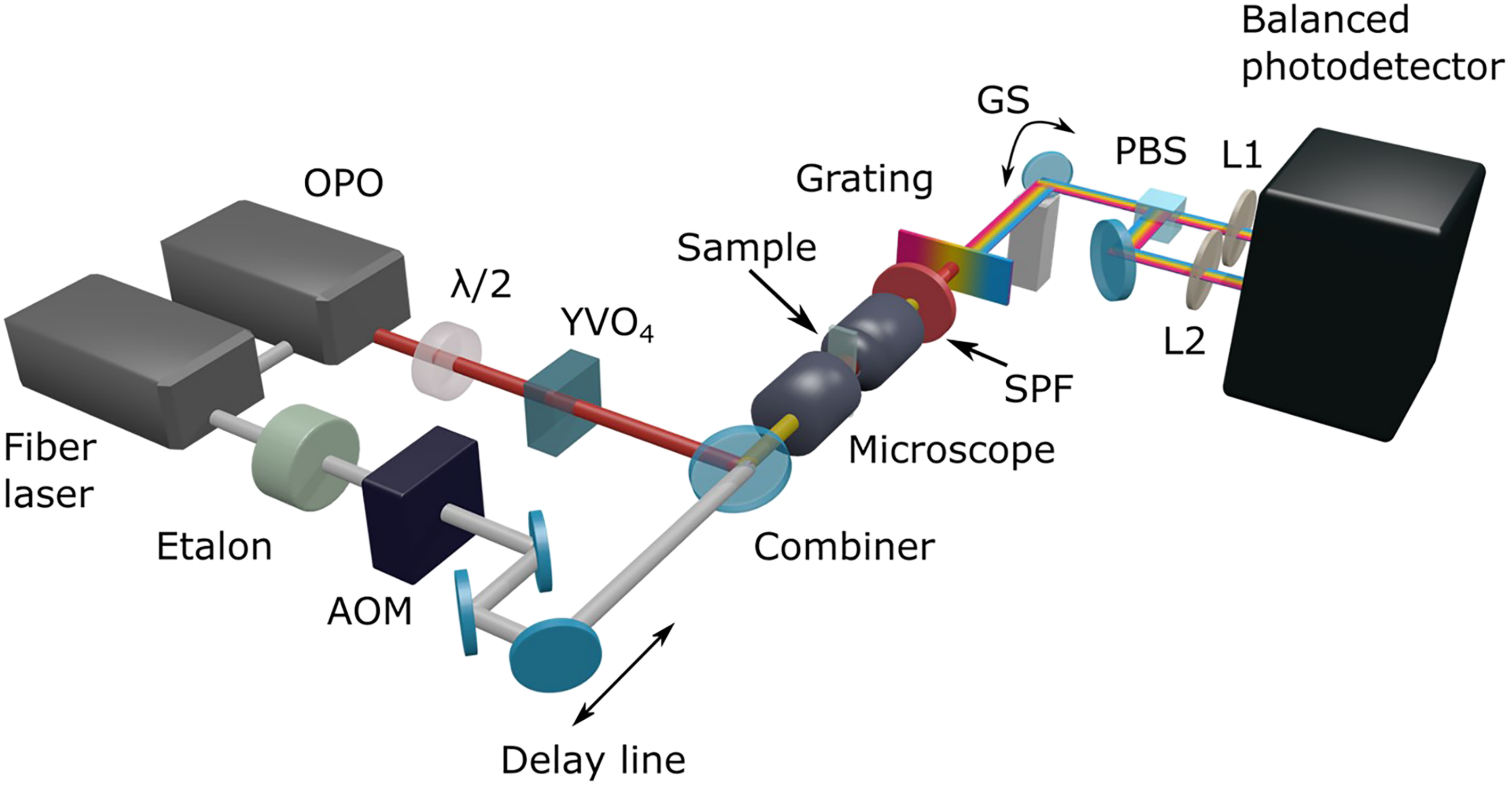
Scheme of the broadband stimulated Raman scattering system. L1 and L2 are spherical lenses both with a focal length of 200 mm. λ/2, half‐wave plate; AOM, acousto‐optic modulator; GS, galvanometric scanning mirror; OPO, optical parametric oscillator; PBS, polarizing beam splitter; SPF, short‐pass filter; YVO_4_, birefringent plate to create the signal and reference pulses

Figure 2 shows a scheme of the broadband SRS setup. The Stokes beam is modulated at high repetition rate (1 to 7 MHz) by an acousto‐optic modulator (AA Opto‐Electronic). Pump and Stokes pulses, both vertically polarized, are temporally synchronized by a mechanical delay line, collinearly combined by a dichroic beam splitter and sent to a home‐built transmission microscope. We employed two confocal plan‐achromat long‐working‐distance objectives with 0.85‐NA, 100× magnification and high transmission in the near infrared (model LCPLN100XIR from Olympus). Before the beam combiner, the pump beam is sent to a half‐wave plate, which rotates its polarization by 45°, and then to a 6‐mm‐thick YVO_4_ birefringent plate, which generates two orthogonally polarized pulses, namely, the reference and the signal, with equal intensity for the IBD scheme.^34^ The signal pulse is synchronized with the Stokes pulse and therefore undergoes SRL. The reference pulse, as it propagates along the fast axis of the birefringent plate, is anticipated by ≈4.9 ps with respect to the signal pulse; therefore, it does not experience SRL, and it serves the purpose to cancel the energy fluctuations of the pump beam. The sample is raster scanned in the focal plane of the microscope with a piezo stage (P‐517.3CL, Physik Instrumente). The average powers incident on the sample are 80 mW for the Stokes beam and 70 mW for the pump/reference beams. Despite the relatively high excitation power, we did not observe photoinduced damage to studied samples. Scanning the beam and spatially filtering each spectral component before the sample, as reported by Ozeki and coworkers,^39^ could further prevent photodamage to sensitive samples, for example, living organisms.

After the sample, the Stokes field is filtered out with a short‐pass filter, whereas the pump beam—still composed by the two collinear and orthogonally polarized replicas—is angularly dispersed by a lithographically patterned transmission diffraction grating (1,850 lines/mm, model T‐1850–800s, LightSmyth Technologies), operating in the Littrow configuration (not shown in the figure for sake of simplicity). The dispersed pump spectrum is further deflected with a mirror mounted on a single‐axis scanning galvo system (GVS011, Thorlabs). The two replicas, that is, the signal and reference, are separated by means of a polarizing beam splitter (CCM1‐PBS252/M, Thorlabs) and focalized by two spherical lenses with focal length *f* onto the two photodiodes of an amplified balanced detector (PDB450A‐AC, Thorlabs). Given the angular dispersion of the signal and reference beams imposed by the grating and the finite diameter *d* of the photodiodes, only a small range of angles Δ*θ ≅ d*/*f* is intercepted by the detectors, which corresponds to a narrow wavelength interval Δ*λ =* Δ*θ*/*D*, where *D* = d*θ*/d*λ* is the grating angular dispersion. For our choice of parameters (*d* = 0.5 mm, *f* = 200 mm, *D* = 2.8 mrad/nm), we obtain Δ*λ* = 1.8 nm which, in the wavelength range of the pump around 800 nm, corresponds to Δ*ν* ≅ 14 cm^−1^, which guarantees sufficient spectral resolution to resolve Raman lines in biological or amorphous samples. By scanning the galvanometric mirror, one can thus vary the detected wavelength and record a high‐resolution SRL spectrum.

In the balanced detector, the two photodiodes feed a differential transimpedance amplifier, which provides an output voltage proportional to the difference between their photocurrents. The output signal is demodulated by a high‐frequency lock‐in amplifier (model HF2LI, Zurich Instruments), whereas the direct current voltage associated to the channel detecting the signal pulse is measured with a high‐performance data acquisition card (NI USB 6341, National Instruments). The demodulated signal is normalized by the direct current voltage to provide the SRL output. Balanced detection allows us to cancel out with the reference pulse the intensity fluctuations of the signal pulse and to approach shot‐noise‐limited detection. The IBD approach has the advantage that the reference is collinear to the signal that crosses the sample in the same position thus experiencing equal attenuation due to local absorption/scattering and passively maintaining the balance during image acquisition without the need for any external feedback.

## 3 RESULTS AND DISCUSSION

**Figure 3:**
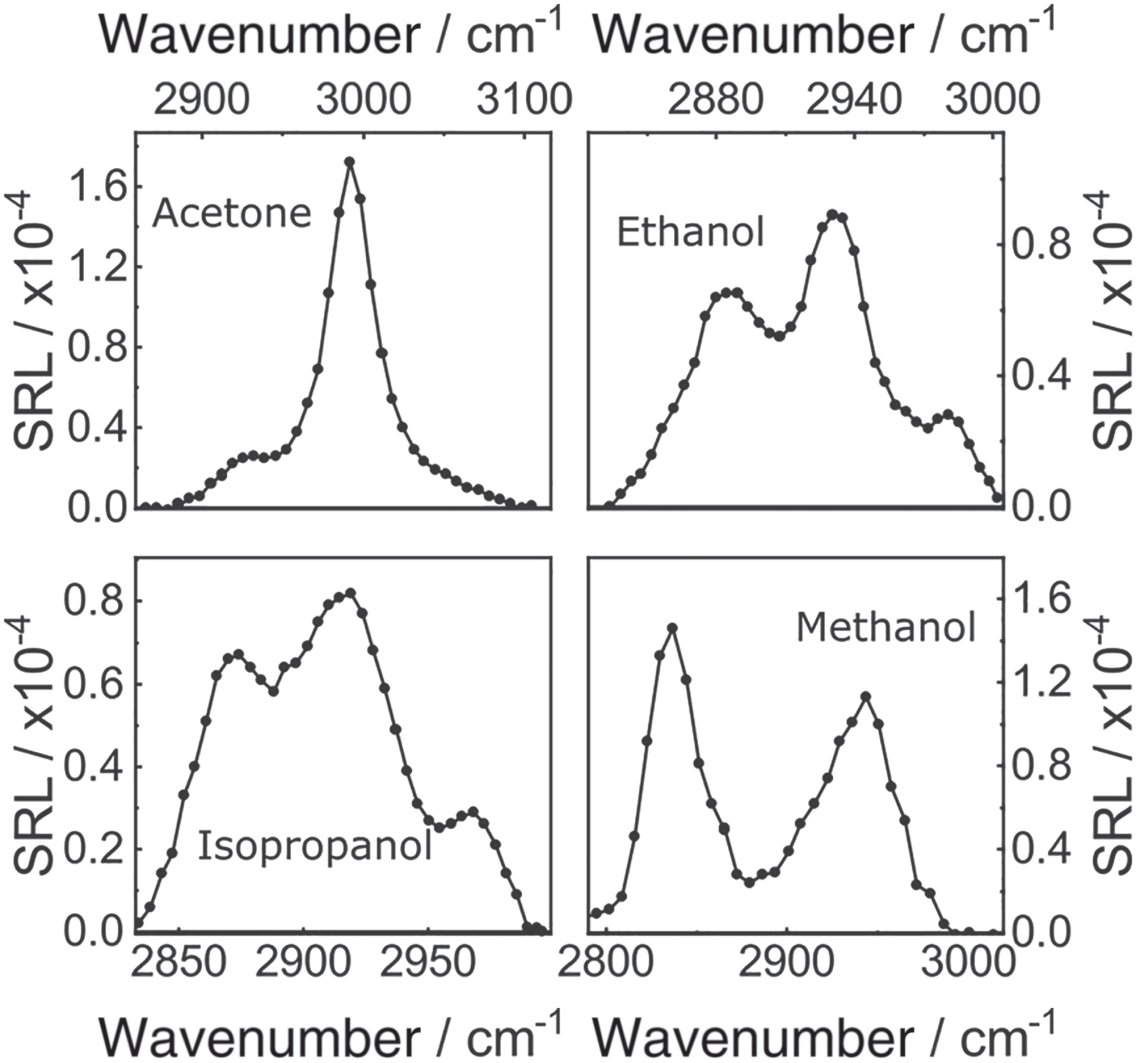
Stimulated Raman loss spectra of reference solvents. The scanning frequency of the galvanometer was fixed at 500 Hz and the integration time of the lock‐in amplifier set to *τ* = 1.8 μs

As a preliminary characterization of the system performance, we measured the SRL spectra of several solvents placed in a 1‐mm‐pathlength cuvette. For these measurements, the focusing and collection objectives were replaced by lenses with 25‐mm focal length. The spectra were calibrated by replacing one of the photodiodes in the balanced detector with a conventional spectrometer and recording a calibration curve of detected wavelength versus deflection angle of the galvo scanner. Figure 3 reports the SRL spectra for acetone, ethanol, isopropanol, and methanol, which are in close agreement with the known Raman spectra of these molecules, in terms of both spectral positions and relative ratios of the different spectral peaks. The good frequency resolution of our setup is highlighted by the ability to distinguish three peaks in isopropanol, which correspond to the CH_3_ symmetric stretch (at 2,885 cm^−1^), the CH stretch (at 2,920 cm^−1^), and the CH_3_ asymmetric stretches (at 2,950 and 2,980 cm^−1^). The spectra were measured at the maximum possible scan frequency of our galvanometric mirror (500 Hz), which corresponds to a 2‐ms acquisition time per spectrum; however, during the scan, the lock‐in time constant was set to its minimum value of 1.8 μs, showing the possibility to reduce the acquisition time by more than one order of magnitude using optimized resonant galvanometric mirrors.

**Figure 4:**
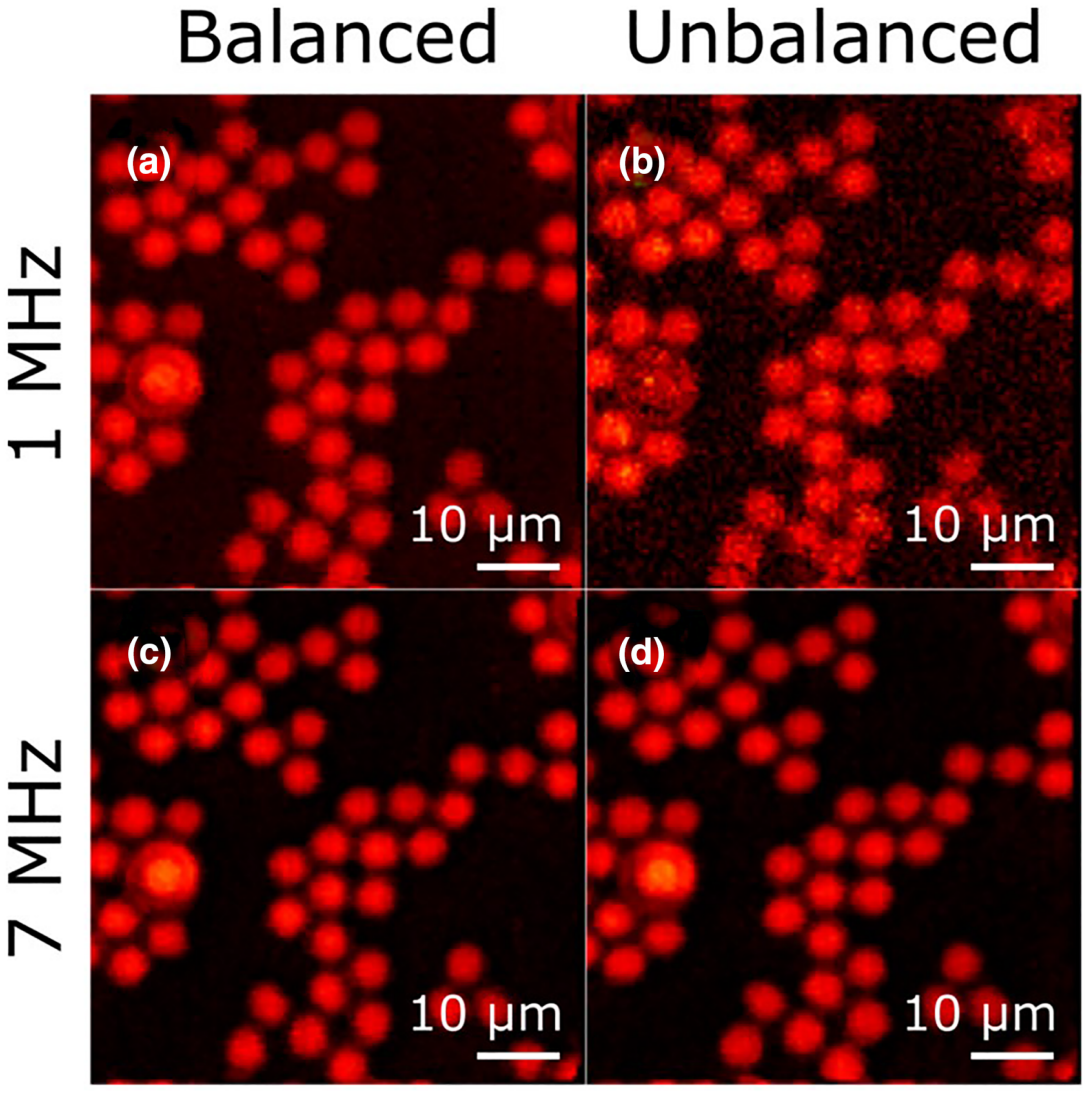
Assessment of the influence of in‐line balanced detection (IBD) on image quality. Panel (a) shows an image of a cluster of polystyrene and poly‐methyl methacrylate beads acquired with IBD at a modulation frequency of 1 MHz, whereas panel (b) shows the same cluster acquired without the IBD. Panels (c) and (d) show images acquired at a 7‐MHz modulation frequency with and without IBD, respectively. Image parameters: pixel dwell time: 10 ms, resolution: 110 × 110 pixels, spectral points acquired per pixel: 50, lo *τ* = 1.8 μs, total image acquisition time: 120 s

The imaging performance of our microscope was characterized measuring a test sample consisting of beads of polystyrene (PS, 10‐μm diameter) and poly‐methyl methacrylate (PMMA, 6‐μm diameter) deposited onto a glass coverslip. We first tested the noise suppression capabilities of IBD by measuring images at the 3,025 cm^‐1^ detuning obtained by keeping fixed the deflection angle of the galvanometric mirror. Figure 4 compares images at 1‐MHz modulation frequency with the IBD turned on (panel a) and off (panel b); one can appreciate an increase in the signal‐to‐noise ratio by a factor of 6.5 due to noise cancelation by the IBD. On the other hand, at a higher modulation frequency of 7 MHz, the increase in signal‐to‐noise ratio is less dramatic for the images with the IBD turned on (panel c) and off (panel d). This can be understood by looking at the RIN curve in Figure 1b, which shows a decrease by 6 dB when increasing the modulation frequency from 1 to 7 MHz, bringing the system close to the shot noise limit. The IBD thus guarantees high sensitivity also in the case of suboptimal performance of the OPO.

**Figure 5:**
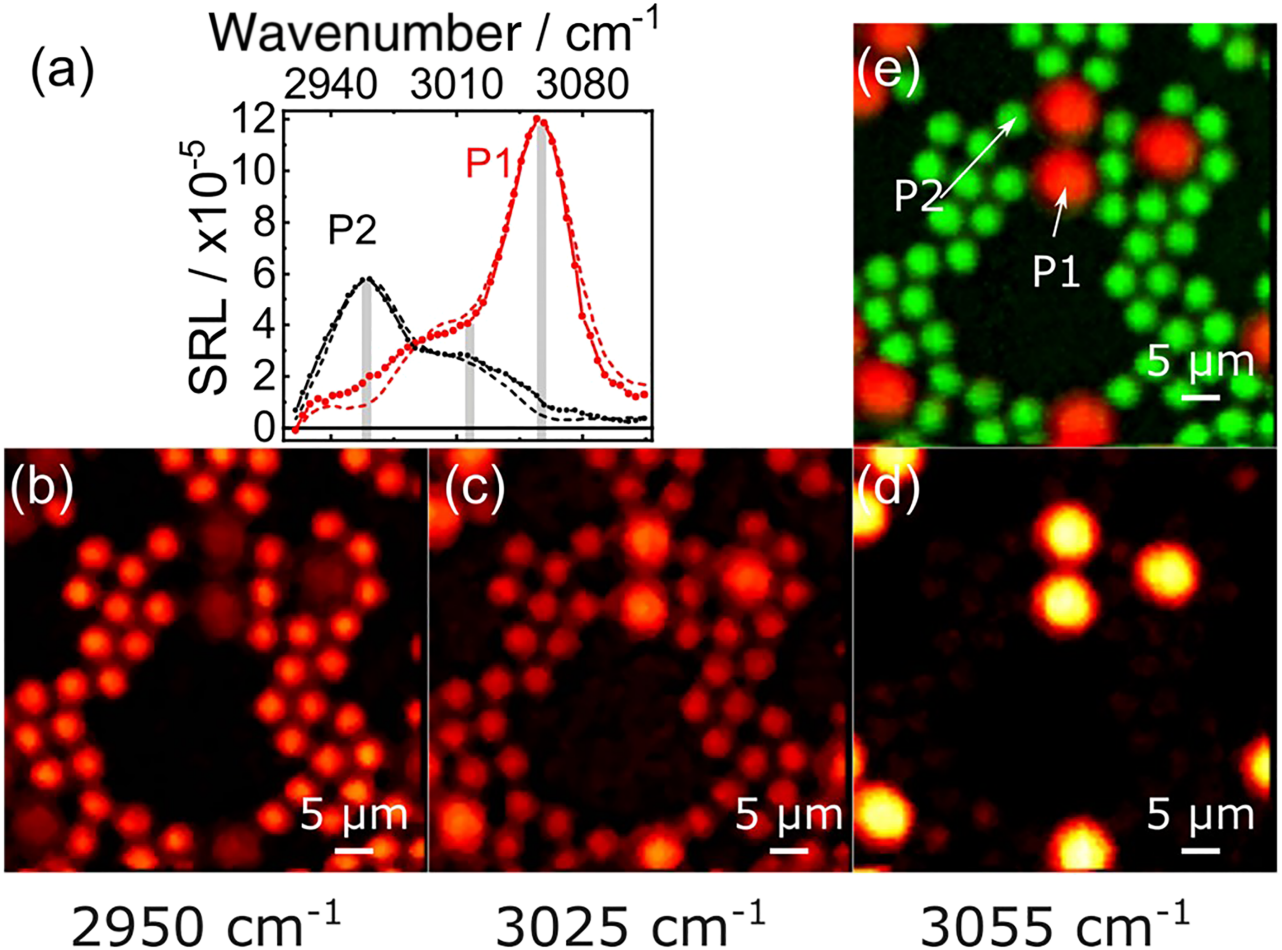
Broadband stimulated Raman scattering imaging of micrometric polystyrene and poly‐methyl methacrylate beads. Panel (a) shows as solid lines and symbols the stimulated Raman loss spectra measured from the beads at the points labeled as P1 and P2 in Panel (e) and as thin dashed lines the two main components of multivariate curve resolution. The gray bars indicate the spectral points used to form the contrast in panels (b–d). Panels (b), (c), and (d) show stimulated Raman loss images at 2,950, 3,025 and 3,055 cm^−1^, respectively. Panel (e) shows the overlay image of poly‐methyl methacrylate (green) and polystyrene (red) concentrations obtained using multivariate curve resolution. Image parameters: pixel dwell time: 10 ms, resolution: 110 × 110 pixels, spectral points acquired per pixel: 50, lock‐in amplifier time constant 1.8 μs, total image acquisition time: 120 s

We next performed broadband SRS imaging by recording, for each pixel of the image, the entire SRL spectrum thus obtaining a hyperspectral data cube. We acquired an image with 110 × 110 pixels and 50 spectral points per pixel which, for a 10‐ms pixel dwell time, resulted in an acquisition time of 120 s for the whole data cube. However, also for this sample, we used a 1.8‐μs time constant for the lock‐in amplifier, which would allow us to increase the speed by at least one order of magnitude using an optimized piezo scanner. From this data set, images at specific wavenumbers can be easily constructed; alternatively, multivariate curve resolution (MCR) methods^40^, ^41^, ^42^ can be applied to the data cube, obtaining images of the spatial distributions of the different principal components identified in the SRL spectra. Solid lines in Figure 5a are SRL spectra recorded at two positions of the sample corresponding to a PS and a PMMA bead (as indicated by “P1” and “P2” labels in Figure 5e), which peak at 3,055 and 2,950 cm^−1^, respectively, in excellent agreement with the known Raman spectra of the compounds. Figure 5b–d shows SRL images at 2,950, 3,025, and 3,055 cm^−1^, which are resonant with the PMMA beads, both beads simultaneously, and the PS beads, respectively. Finally, Figure 5e shows a concentration image of the two principal components identified by MCR, whose spectra (dashed lines in Figure 5a) match those of PMMA (P2, green) and PS (P1, red).^43^

**Figure 6:**
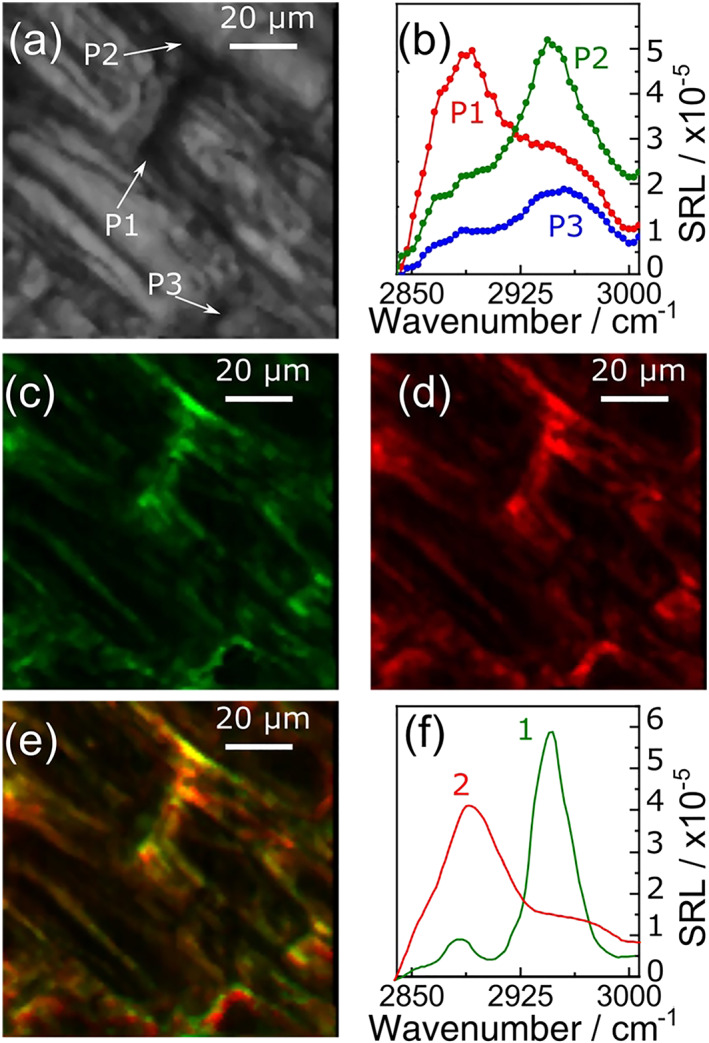
Chemical imaging of a leaf of an *Elodea Canadensis*. (a) Linear transmission image, (b) stimulated Raman loss spectra at the points labeled as P1, P2, and P3 and indicated with the arrows in panel (a); panels (c) and (d) are SRL images at 2,890 and 2,947 cm^−1^, respectively; (e) the overlay image of the concentration distributions of the two main components of MCR; (f) corresponding spectra. Image parameters: pixel dwell time: 10 ms, resolution: 110 × 110 pixels, spectral points acquired per pixel: 50, lock‐in amplifier *τ* = 50 μs, total image acquisition time: 120 s

To validate the applicability of the system to the study of biological samples, we acquired broadband SRL images of a thin cross section of the leaf of the aquatic plant *Elodea Canadensis*, sandwiched with water between two microscope slides. Figure 6a reports the linear transmission image of the sample; one can clearly distinguish the typical plant cells with rectangular shape and the dark cell walls with lower transmission. SRL spectra at selected points of the sample are reported in Figure 6b, showing significant variations from one point to the other. SRL images at 2,890 and 2,947 cm^−1^ are shown in Figure 6c,d, respectively. Strong SRL signal in the C─H stretching band (2,850–3,000 cm^−1^) is observed within the walls of the plant cells, as it can be seen by comparing these images with Figure 6a. Similarly, to what was observed and discussed in Crisafi et al.,^34^ we do not register any appreciable birefringence/anisotropy of the sample, which could cause a depolarization of the beams during propagation and thus a reduction of the IBD performances. Differently from the previous sample, which consisted of two chemical species (PMMA and PS) well separated in space, the eukaryotic cells are highly heterogeneous so that the SRL signal results from the superposition of all the molecular components contained in the focal spot of the pump/Stokes beams, which may be present simultaneously at different concentrations. Therefore, chemometric approaches^44^, ^45^, ^46^ such as MCR are required in order to extract the spectra and the spatially dependent concentrations of the species contained in the sample. By performing MCR on the data cube, two main species were identified, whose spectra are reported in Figure 6f. By comparison with Raman spectra of plant constituents, they closely match the known spectra of cellulose (red solid line, peaking at 2,880 cm^−1^) and pectin (green solid line, peaking at 2,950 cm^−1^), which are the main constituents of the cell walls.^47^ Figure 6e shows a composite map of the concentrations of these two species in the plant cell walls, demonstrating the capability of our broadband SRS microscope to identify components in a heterogeneous system.

Our broadband SRS system does not yet reach record‐breaking performance in terms of acquisition speed; however, it offers ample room for optimization and presents some advantages with respect to competing systems, as discussed in the following. Hyperspectral SRS systems typically tune the emission frequency of one of the synchronized narrowband laser systems by an intracavity element, which may result in wavelength‐dependent variations of the laser stability and/or repetition rate or absolute timing of the frequency‐scanned pulse; our approach avoids these issues by scanning the detection frequency inside a fixed broadband pulse after the sample. The SF technique achieves very rapid spectral tuning by changing the delay between two strongly chirped pulses; however, it is challenging to extend the detection bandwidth beyond ≈200 cm^−1^, due to the reduced temporal overlap between pump and Stokes pulses as their delay is changed, which decreases the strength of the nonlinear signal on the tails of the tuning range. Our approach, on the other hand, is not limited in detection bandwidth. For multiplex SRS systems, the best performance was obtained by Liao and coworkers using an array of 16 tuned amplifiers for simultaneous detection of SRS signal at 16 frequencies. This frequency sampling may be too coarse for some applications, whereas our approach has frequency resolution only limited by the bandwidth of the narrowband Stokes pulse. If needed, one could employ an etalon with narrower linewidth and either a photodiode with a smaller diameter or a longer focal length in detection to reach 10 cm^−1^ resolution, which is the ultimate spectral resolution required in vibrational spectroscopy of solid‐state samples.

## 4 CONCLUSIONS

In this paper, we demonstrated a novel approach to broadband SRS microscopy, which is a hybrid between hyperspectral and multiplex detection. In our system, a narrowband Stokes pulse, obtained by spectrally filtering a commercial femtosecond Yb:fiber laser, is temporally synchronized to a broadband pump pulse, generated by a home‐made femtosecond OPO. After the sample, the pump pulse is angularly dispersed by a diffraction grating, and the SRL spectrum is measured for each pixel of the image by scanning the pump–beam deflection with a galvanometric mirror. In‐line balanced detection is used to reduce laser fluctuations and bring the sensitivity close to the shot‐noise limit. Our setup allowed us to accurately measure the SRS spectra of several solvents and to obtain broadband SRS images of polymer beads test samples as well as of the distribution of different biological substances within plant cell walls.

Our system is simple, as it does not require sophisticated electronic detection chains, and can be straightforwardly applied to commercial laser systems consisting of an Yb:laser or Ti:sapphire pumped femtosecond OPO. Currently, the acquisition time of a single SRS spectrum is limited to 2 ms by the 500‐Hz maximum scan rate of the employed galvanometric mirror, resulting in acquisition times of the order of minutes for one hyperspectral cube. However, the sensitivity of our apparatus would allow considerably higher scan rates, which are possible using resonant galvanometric mirrors. Alternatively, one could use an approach similar to the one demonstrated by Ozeki and coworkers,^48^ where the hyperspectral cube is built by stacking images acquired at different detection wavelengths. In the latter case, one should use a hybrid scanning scheme consisting of a sample‐scan stage in the direction of the beam dispersion, to keep this optical path fixed, not to alter the spectral resolution, and a single‐axis galvanometric mirror scanner in the direction perpendicular to the dispersion plane, similarly to what have been done by Liao and co‐workers.^31^
